# Gender and Environment in the Interior of Santiago Island/Cape Verde: Sand Harvesting From Women Heads of Families**

**DOI:** 10.3389/fsoc.2021.625405

**Published:** 2021-04-22

**Authors:** Miriam Steffen Vieira, Eufémia Vicente Rocha

**Affiliations:** ^1^ University of de Rio dos Sinos Valley (Unisinos), São Leopoldo, Brazil; ^2^ University of Cape Verde (Uni-CV), Praia, Cape Verde

**Keywords:** gender, cape verde, women, environmentalism, development

## Abstract

Cape Verde is an island country and Sahelian, where the climatic conditions cause a rainfall deficit originating dry periods causing a fragile agricultural development. The rural world is facing various problems such as lack of land for cultivation, lack of water and soil erosion. The "*apanha de areia*" (sand haversting) refers to the extraction of sand and gravel from the sea and rocks. Although it is considered as an environmental crime, the activity is carried for generations and supplies the civil construction business of the country. This study analyze this activity from the perspective of women from the interior of Santiago, in the locality of Charco, in the municipality of Santa Catarina. The research was carried out based on an ethnography of long duration, with spaced field visits, since January 2009 and the monitoring of environmental and gender policies in Cape Verde. As results, we highlight women's agency in the face of a context of growing social inequalities.

## Introduction

Cape Verde is commonly presented in the literature as an island and Sahelian country, in which its climatic conditions cause a rainfall deficit, originating dry periods that persevere and, therefore, impose a fragile agricultural development. As a result, poverty and vulnerability are phenomena that have crossed the archipelago’s entire history.

The Cape Verde archipelago is located on the West African coast. It is composed of ten islands, nine of which are inhabited, being its population comprised of 248,280 women and 243,403 men, according to the 2010 population census. (Instituto Nacional Estatística, 2011). Considered as a medium developing country, the percentage of the poor population (people living below the poverty line, based on an income of less than 49,485$00 per year) was 26.6% in 2007, according to Cape Verde’s Unified Questionnaire of Basic Wellbeing Indicators, published in the Statistical Yearbook. (Instituto Nacional de Estatística, 2015, p. 63). Regarding the poverty distribution, the same survey showed a higher incidence in the rural areas, indicating that “the depth of poverty was 8.1%, whereas in urban areas this value was 3.3%, and in rural areas, it was 14.3%” and, concerning the intensity of poverty, “it reached 3.4% in 2007, while in urban areas it was 1.3%, and in rural areas 6.3%”. (Instituto Nacional de Estatística, 2015, p. 63).

However, the poverty concept does not only cover income and consumption. As an institutional category that supports public policies, to beyond the deprivation of elementary needs, the notion of poverty became part of the deprivation of freedom to choose, to outline an earlier situation and, consequently, the non-participation in the policy-making processes. It is a multiple deprivation, that is, a deprivation in several domains. In this case, one can speak of exclusion, even though the two concepts–poverty and social exclusion–present distinct realities that cannot always coexist, although they are often confused and linked to each other.

Pedro Demo, (1995) talks about two different types of poverty: the socioeconomic and the political. In his perspective, when we think about poverty, the material aspect is the first one that comes to mind; it is a deficiency. However, the sociologist recognizes a type of poverty associated with the field of power, that is, a dimension of citizenship, participation, and forms of political organization. All of this is achieved when the poor begin to reflect on their condition and look for solutions to break with this influential logic; when they cease to be mere objects and do not see poverty as something innate.

In the 1990s, the Cape Verdean governments started to include the poverty dimension in their policies. In 1997, the National Program to Fight Poverty (PNLP) was presented as a decentralized and participation tool for the different social actors involved, considering that the fight against poverty also came to be understood as a task for the poor themselves. This effort to reduce and eradicate poverty began to rely on both macro and microeconomic policies, making it clear that the intention was to present “growth with inclusion, growth with a human face”, as documented in the Program to Fight Poverty in Rural Areas (2008–2011). These characteristics have been pointed out in Furtado’s research (2008) on the development of policies concerning poverty in Cape Verde and in Veríssimo’s study (2015) on the implementation of the Program to Fight Poverty in Rural Santiago.

Considering that poverty is constituted and distributed in different ways, depending on the islands and municipalities, and being more predominant among the rural population, it is noteworthy that the islands that mostly comprise rural inhabitants are the most affected, being Santiago Island one of them. According to (Furtado, 2008, p. 21):

“Natural, technical and social conditions of agricultural production, a land structure based mainly on the indirect exploitation of land and smallholdings, rudimentary production techniques and technologies in rainfed agriculture characterize the world of Cape Verdean agricultural production, making productivity extremely low and the income earned not able to guarantee minimally the survival of families, making more than two thirds of the farm members to have sources of income from extra-agricultural activities.”

Thus, the rural world faces several problems related to the lack of land for cultivation, besides other environmental phenomena such as insufficient water for consumption and agricultural use. Regarding the water consumption in Cape Verde, it is mainly from underground sources. Its flow type largely regulates the exploitation of surface water that has a torrential origin. Therefore, its quantity is underutilized due to the difficulties of capturing it.

The water issue is considered of vital importance to overcome barriers to development. This is the result of the countless efforts to build water infrastructures able to solve the problem of water shortage in Cape Verde, such as the dams. (Ferreira, 2016).

In these policies, it is assumed a development that bets on a multidimensional character, on an interdisciplinary approach, in which different dimensions of life are articulated, demanding participatory and empowerment methodologies. As Chambers, (1983) has shown, farmers are expected to become “architects and actors of their own development.”

According to the Poverty Reduction and Growth Strategy Document III (DECRP III 2012–2016), poverty in Cape Verde is considered rural and female:

“Concerning the different impacts in each population group, poverty in Cape Verde has been a historical problem that takes into account the weaknesses and vulnerabilities faced by the nation. Poverty tends to be rural, and a higher proportion of women are poor if compared to men. The same is true for female-headed households compared to male ones. Poverty in Cape Verde is considered then rural and female (DECRP III, 2012–2016).”

In this context, the aim of this study present some reflections from a long-lasting ethnography, which started in January 2009, based on the perspective of women from Charco, located in Santa Catarina municipality. Therefore, we carry out field observations, interviews and informal conversations. All testimonies and narratives collected during fieldwork are using fictitious names to preserve identities (Beaud and Weber, 2007; Fonseca, 2010).

## Charco’s Region

Santiago Island is the largest in terms of surface (991 km^2^) and concentrates more than half of the country's total population (approximately 54%). Santa Catarina is one of its nine municipalities and, in the past, was the most populous one. Today, agriculture and livestock are still vital activities for the region.

For this research, we chose the Vila de Ribeira da Barca, a coastal region in Santa Catarina municipality, and other zones in its large area, particularly in the Charco, which is a region considered strongly impacted by the “*apanha da areia*” (sand harvesting). Sand harvesting is a process that consists of the extraction of sea sand and rocks, such as gravel and crushed stone, for the use in civil construction. According to Moassab and Vieira (2016, p. 55),

“Territorial restrictions added to the water shortage result in high pressure on the main natural resources which, as well as the lack of employment, are at the basis of the economic activities that use aggregate extraction.”

The Charco’s drainage basin extends over an area of 35.58 km^2^ with a perimeter of 32 km. Its annual average rainfall is 400 mm/year in the high-altitude spaces and 150 mm/year in the low. Besides that, it presents climatic extremes that diversify from the sub-humid, semi-arid to the arid, promoting three agroecological zones. The basin areas contiguous to the sea today face problems of marine intrusion. Hence the soil salinization, which, according to Cape Verdean technicians, is the result of the exploitation of aggregates (sand harvesting) by the local and surrounding populations.

Charco’s community, which is composed of 266 inhabitants, including 144 women and 122 men, comprises the areas of Figueira Coxo, Covão Dentro, Djangago, Lém Freire, Terra Vermelha, Taberna, and Locale (Dogoule). In them, a total of 51 households were identified, being 30 of them female-headed families, according to data collected from the Third General Population and Housing Census in 2010. (Instituto Nacional Estatística, 2011).

However, at first, this was not what called our attention concerning the Charco’s community. It was, on the one hand, the evidence of an intense movement of women dedicating themselves to sand harvesting–something that came from decades ago –, and, on the other, the institutional statements that emerged in this scenario focusing on environmental degradation and, consequently, on the women’s culpability. Based on this, the need to (re)convert women to agriculture or to other income-generating activities appears, with government and/or non-government support.

Regarding policies, in addition to the above-mentioned program aimed at reducing poverty, Environmental Action Plans (PANA) were also developed. The first, for the period from 1994 to 2004 (PANA I) and, later, covering the period from 2004 to 2014 (PANA II). The second plan was composed of six volumes, being the first one dedicated to “the impact of aggregates harvesting and extraction”. Among other environmental damages, this activity is considered to be responsible for “landscape degradation”, impacting the development of tourism. Then, in 2007, the aggregate extraction without authorization was criminalized.

Thus, it is not by chance that poverty and social exclusion have become political problems, since the populations’ conditions of existence, as well as their laws and rights, are at stake; they are not only enactments. On the contrary, its effectiveness legitimizes democracy. In a democratic context, decisions must go through the target audience of certain policies or programs and recognizing them as social actors in these environments is the way to guarantee participation in decision-making processes (Oliveira, 2000). This dynamic viability in a Cape Verdean rural community makes us reflect on the concept of *dialogic ethics* of Roberto Cardoso Oliveira, (2000), considering that, for the author, the issue involves a democratic process, a process that must be guided by symmetry, based on the understanding of those who are touched by a discourse. Everyone must be part of the same space of participation and intervention.

What Mr. Sogni, a resident of Ribeira da Barca, assured us is that “out of necessity, there is no more sand, because people took everything to harvest, to sell, to live”. This statement allowed us to identify a hierarchy among the residents of these communities. The women who participate in the sand harvesting complain that they are despised, they are insulted of long necks, and are widely criticized. In this case, they insist on showing that, on the one hand, there are women who participate in this process, and for this reason, they show signs of a daily struggle; on the other hand, other people show up, women and men, who do nothing and only expect to receive or simply wait for the others to give them something.

In this context, sand harvesting reveals agency and contestation dimensions in relation to the situation of social and economic inequality of women and families in rural areas.

More recently, in 2015, the non-governmental organization *Renascença Africana*: West African Women's Association (RAMAO) in Cape Verde held public sessions to create awareness in the population of several islands where extractive activities were manifested. It is a project that involves other countries in the African subregion and aims at “preventing risk in coastal areas”. The first action was in Santiago Island north side, comprising the municipalities of Tarrafal, São Miguel, Santa Cruze, Santa Catarina, therefore, covering women from the Charco’s community.

A television news broadcast about this awareness-raising action presented the perspective of women who participated in this meeting. A group of women who are in the sand harvesting made a manifest entitled "*lenço branco na cabeça*" (white scarf on the head). One of the ladies said that sand harvesting “harms our health first, then it harms the environment; but as we have no other option, we keep going there”. Another lady talked about the difficulties of supporting her family–one of her children was disabled, so the extraction was what was left to her.

We participated in RAMAO’s second action, held on July 11, in the Old City, contemplating Santiago Island south side. The meeting entitled “Risk Prevention in the Coastal Areas of Santiago Island” was attended by women from different locations, many accompanied by their children. The event took place during the morning and ended in a community lunch and a presentation of *batuko* ^1^ *.* It was also present authorities in gender policies, representatives of the Cape-Verdean Institute for Gender Equality and Equity (ICIEG) and UN Women in Cape Verde, researchers from the Center for Research and Training in Gender and Family at the University of Cape Verde (CIGEF), as well as government representatives from the environmental field. The event started with two technical training courses on environmental protection and risks arising from extractive practices conducted by the population. Participants were given sheets of paper and pens for taking notes. In the end, RAMAO’s president opened the floor to the women: “Now, you tell us what to do. How can we find another solution together, so that in the future we won’t be without beaches, without agriculture? Now, it’s up to you. Speak up, please.”

After an initial silence, many and many testimonies from women who have been in the sand harvesting for 6, 15, and 34 years have emerged in sequence: “I’m over 23 years in the sand, my life is in the sand”; “In our life, it’s that sand that is worth it; there's nothing we can do"; "We have to extract it, otherwise we won’t survive"; “I’m the mother and father of my children”; “I’m the head of my family”; “I’ve been working in this since I was fifteen and I had a child”; "I’m 34 years in the sand, my husband died 29 years ago and left me with six children."

Lastly, when asked about possible alternatives to stop the sand harvesting, a lady did not hesitate to criticize palliative measures, referring to income generation programs from animal husbandry. She said: “I have to do it to feed the piglets!^2^.”

These events make the women’s place of speech explicit, highlighting the overload of work to which they are exposed for their family reproduction, the State’s fragility for the provision of care services, as well as job opportunities with decent conditions for the survival of their families.

### *Maré Ta Kunpanha Lua* ^3^: Nature, Gender and Work

The first house we visited was close to the sea, on the slope where it starts the Lém Rocha area. Next to the entrance door, there is a square space filled with sand. We didn’t ask, but afterward talking to Tê, she explained that after January 8, they could no longer extract sand. However, the sand that was off the sea edge, which was already extracted, could be used. In this way, we saw some piles close to the residences. It was interesting to see its usage; it didn’t seem to be for sale. House repairs, maybe? Many were under construction.
In front of the house, there was a boat built by Mr. Davi, a 70-year-old fisherman that only worked in fishing from time to time. We talked to him over lunch.
From the house, we saw children playing in the sea, young people playing football, small trucks heading to Charco with some people on the top, and many women and children passing towards the Charco, with buckets on their heads and water–what Eufémia designated as a Pilgrimage (Field notes, Ribeira da Barca, 02/20/2010).

**FIGURE 1:**
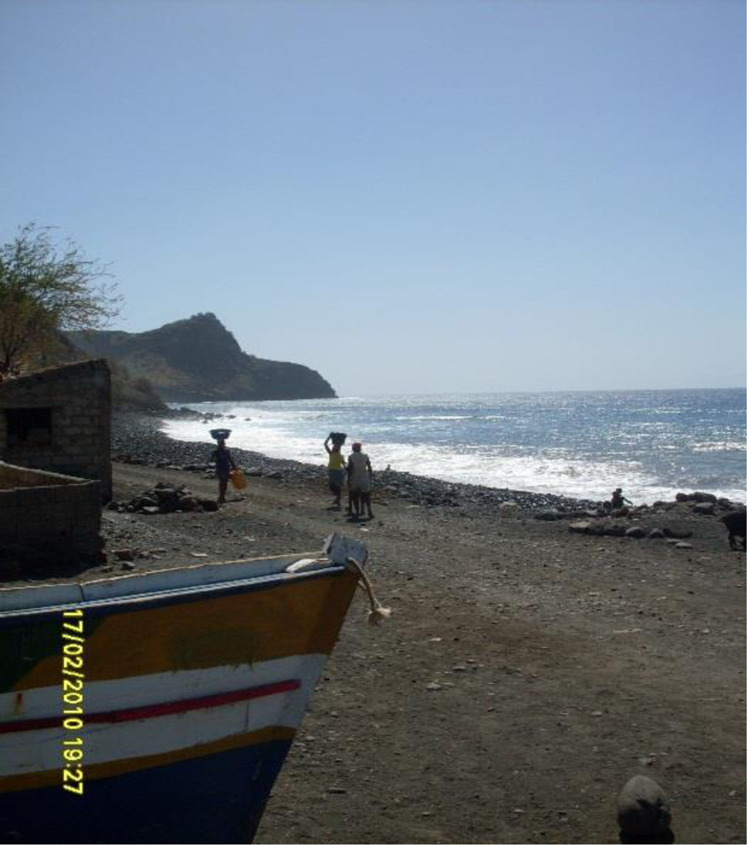
Pathway from Ribeira da Barca to the Charco. Source: Images taken by the researchers, February 2010.

**FIGURE 2:**
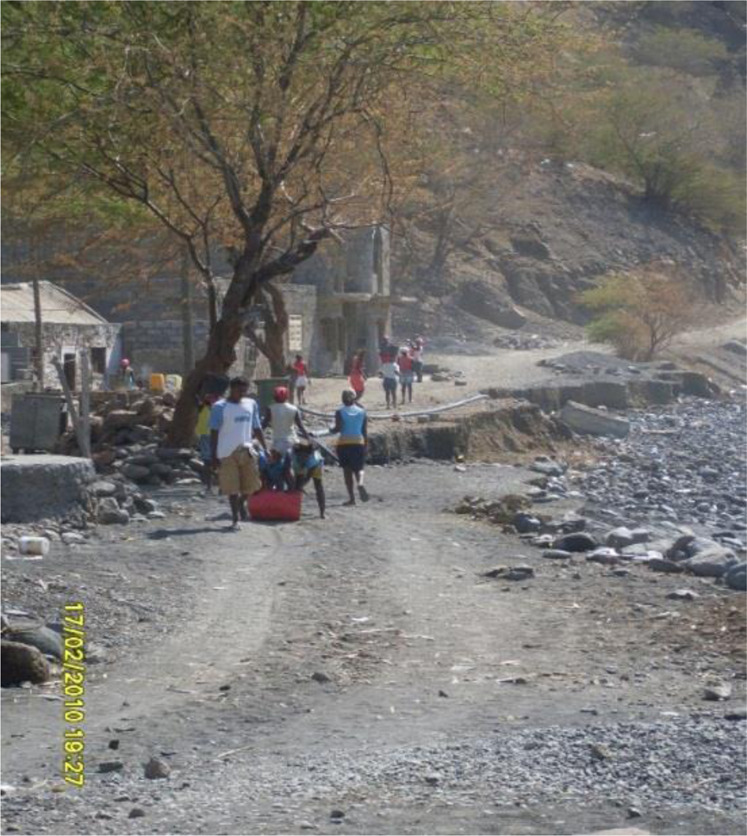
Pathway from Ribeira da Barca to the Charco. Source: Images taken by the researchers, February 2010.

**FIGURE 3:**
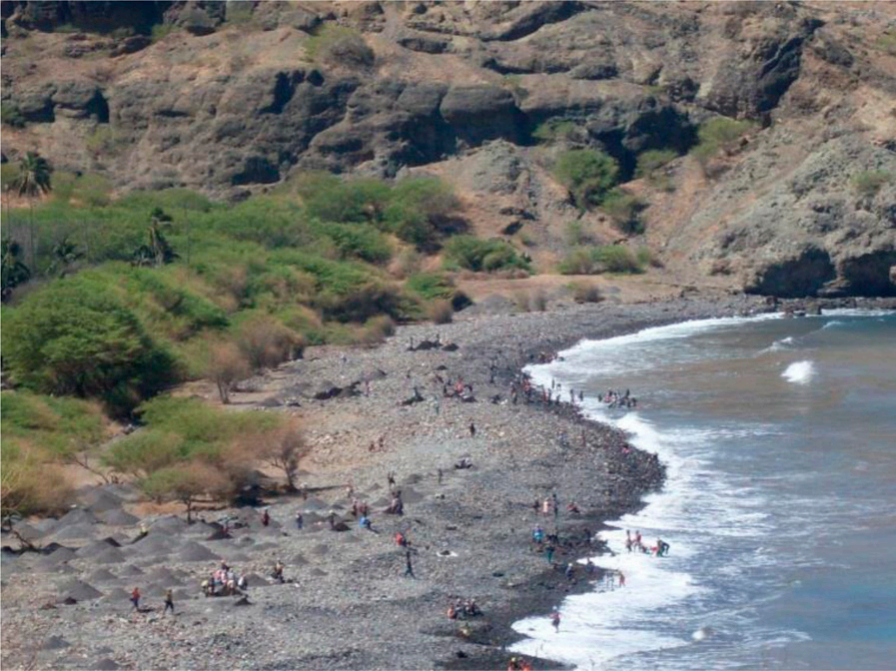
Sand harvesting, Djangago Beach/Charco. Source: Images taken by the researchers, March 2010.

When we talked to some young women about the pace at which they filled the *galuchos*, name locally used for the vehicles that transport aggregate materials, they said that everything depended. It would not be possible to know, since it was the sea that mainly dictated the rules. Per day, they could fill one *galucho*, or even more, if the tide was good. When the sea was rough, it was too dangerous; they avoided going into the sea and going further to not run the risk of getting hurt on the slippery rocks or even suffering other accidents. Therefore, the best time for the sand harvesting was when the tide was going out. It was then when we observed the pilgrimage, a women’s group moving together, carrying their bucket and other utensils–scarf, *sulada* ^4^, *unheira* ^5^– towards Djangago beach, in the Charco, with some heavy vehicles. Women who once were children and even in this life cycle experienced the sand harvesting, as Beta insisted, referring to the fact that they dedicated themselves to this activity as children, during their school vacations and holidays: “each one has its own weight, each one gets what they can!” (Field notes, Ribeira da Barca, 02/2010) (See Figure 1, Figure 2 and Figure 3).

Mr. Sogni, Beta's father, made a point of explaining to us about the moon's interference in the sea. They followed each other. When the moon was high above our heads, it meant that the tide was dry or low. When the moon was rising or setting, the tide was full or high. He also said that the sea changed every 40 or 45 h.

This relationship between the sea and the moon was significant for the construction of their fishing boats, given that cutting the wood for this purpose was closely related to the tide, i.e., the wood would be good and dry for cutting and manufacturing the boat if the tide was, likewise, dry.

Therefore, the rhythm of work is intertwined with the rhythms of nature, that is, the sea is an important guide on how the work will be conducted and allows a greater autonomy in its management. Sand harvesting depends not only on the sea conditions but also on domestic tasks, illness, and/or other situations that may come into play. As Beta explains, a young woman who is dedicated to this activity of extracting sand and gravel, these women constitute societies. Her 56 year-old mother and her, along with two other women, are partners; they all live in Ribeira da Barca and go to the Charco to work.

Ane, Beta's sister, exemplified to us that among her sister, their partners, and her there are no conflicts in general, but in other groups there are. Sometimes, a partner may not go to work one day, and the others determine, by mutual agreement, whether she will be paid for that day or not. In their case, she says: “I may not go one day, I may have clothes to wash, to iron and I know that Beta will work for me. I know I can get sick and they will work for me, but we will share the money. But I know that in other groups it doesn’t work like this, that is why the discussions begin ”(Field notes, Ane, Ribeira da Barca, 2010). For this reason, we observe changes and adjustments in the composition of the teams. These tensions, however, did not arise only between women, but also between them and the vehicle drivers, who usually buy the aggregates. This happens for different reasons: 1) they can take months to pay; 2) they may not pay; and 3) if they have a specific clientele, they are loyal enough to buy sand only from a few teams, making the rest of them unhappy.

In this perspective, these arrangements seem to be more flexible in relation to the labor market, presenting agreements between the team members, agreements between the teams themselves, with the flexibility to manage the working hours and to support work in networks in which the presence of families, mainly female-headed, is the base.

This flexibility also arises in other situations, when, for example, Mr. Sogni told us that his youngest son, who attended his first year at the University of Santiago, did not obtain a subsidy to support his studies through the Cape Verdean Foundation for Social Action in Schools (FICASE) and had to work in the sand since only fishing was not enough to support all his education expenses. Until the end of 2015, we learned that this young man finished his degree, but, because he did not get a job, he remained working with fishing and sand harvesting.

Although the sand harvesting is recognized locally as a work that involves more women than men, men are also increasingly present in this activity, but only in some circumstances, as two partners who were on their way to the Charco explained to us. These 22 and 27 year-old young men identified themselves as fishermen; they said that they prefer to go fishing, but as the sea was rough, they would have to go sand harvesting because they needed some money to endure until the end of the year and it would not be possible to wait for the sea conditions to improve.

However, it is not just the nature conditions that sets up the pace of Charco’s life. Mr. Sogni regrets that “today there is no more fish!”. He explains to us about the Cape Verde government agreements with foreign countries that allow the entrance of fishing vessels and about the impotence of traditional fishing techniques compared to theirs: “we caught a tuna, chewed it and spit in the sea, it was a bait! Fishing was done using line. But foreign boats have greater capacity, fishing is in a greater quantity, so there are no fish in Cape Verde!” (Field notes, Sogni, Ribeira da Barca, 02/2010)^6^.

This phenomenon was also observed by Gonçalves, (2016) in a study on artisanal fishing in Cidade Velha, in a context of restrictions faced by the patrimonialization of the space, as well as environmental regulations and international agreements in this field.

Regarding the participation of young people in the sand harvesting, the current President of the Association of Fishermen of Ribeira da Barca^7^ stated that even with the secondary education completed, due to the lack of work, people still dedicated themselves to this activity. Recognizing that this problem should be addressed by the government, he considered that the Association should encourage awareness-raising actions against the sand harvesting since it is better for the general population, which can achieve greater benefits from tourism. However, he pointed out some difficulties at this moment: first, he explained that, as this activity progresses, it makes more difficult for turtles to spawn on the beaches and, consequently, the possibility of these animals disappear from the marine biodiversity increases. Second, he says that the extraction also affects fish reproduction, given that women go into the sea and stop when the water reaches their necks. Third, that there are numerous complaints in the Capitania dos Portos by the Charco’s residents and farmers, portraying their land salinization and the infiltration of seawater itself into fresh/drinking water springs or wells, making it unhealthy. Fourth, he mentions the invasion of the sea to the point of jeopardizing the houses and properties closest to the beaches since sand and gravel are no longer there to play their natural protective role. Finally, he criticizes the authorities for not exercising its functions, i.e., there is a bad control regarding this issue because there is no inspection concerning the sand harvesting.

However, over the years, we have observed the increase of discourses and actions by state institutions in regard to the extraction of aggregates through the press, on Santiago Island, and in other islands of the archipelago. As an example, on May 15, 2010, the online vehicle *A Semana* reported: “The General Directorate for the Environment (DGA) manifests against the sand harvesting on São Filipe beaches” on Fogo Island. For this reason, it would be proposed to the government to suspend the aggregates extraction on these beaches to avoid an “environmental disaster” (Centeio, 2010). More recently, in 2014, this phenomenon was seen on São Nicolau Island (Pina, 2014), as well as resistance by extractors on São Vicente Island.

Similarly, studies on the impacts of the extraction of aggregates and legal provisions for their regulation became recurrent. We highlight, in this regard, a news from the beginning of 2016 that points out the need for a new instrument to regulate the extraction activity and the inspection intensification in some parts of the country, including Ribeira da Barca. This municipality is alluded to as “a special case” for some of the reasons set out by the associative leader above-mentioned, but this time through the words of the current National Director for the Environment, who pointed out the following:

“Only dialogues are not being enough and within the short term, we will have to take more radical measures. In the near future, we will have to articulate with the National Police and eventually with the Armed Forces to enter the community and make people comply with the legal obligation. Sand extraction in Cape Verde is prohibited by law and it makes no sense for people to be making excuses of any kind (RETOMA, 2016).”

### Land and Water Seen as Male References

These women are “in the middle” of two large property families, who are also frequently in a dispute over land and water. Therefore, considering that these families have been exploiting these assets in the area since before the country's independence, the priority remains theirs at these women’s expenses. According to the Brianda Norte Association’s president, who has been helping the community and these women in an attempt to make their pieces of land profitable in these cases, these ladies are always at disadvantage.

The advantage always remains on the strongest side, and despite the changing situation, they remain the strongest. Exactly, this is a problem that we essentially have, that water, as you could see, falls into the tank up there flooding It is very deep; it consumes more than 4 tons of water. So, today we do not have water for this type of [flooding] agriculture, that is the only problem that we have, the only one. It is two things; [more] the sand harvesting, but there is no policy for that. Those people who have the water, even if the water is there they don't want you to mess with it. Water has become like an asset, but theirs [because] they inherited it, it is private. (Field notes, Donan, Charco, 04/2015).

Over the years that we have been following these women in the Charco’s community, the water issue became increasingly present. Due to the lack of this good, in 2015 these women were already starting to lose hope that one day they would be able to work in the piece of land they had won, as a policy of converting sand harvesting to agriculture. Even considering that they had so far failed to install a modern irrigation system, they said that they could at least carry the water on their heads. However, the two large landowners did not allow them to do even this.

This associative leader showed us during the different meetings and dialogues we had that this is a dispute that does not dissociate one good from another. Considering that large landowners have always exploited these lands, water ownership has never been called into question. It was also part of the “package”.

We visited the community at different moments, and sometimes the panorama was bleak. Lots of water wasted on lands that were not even in use, using flooding, a traditional system in the agriculture practices of Cape Verde, which has fallen into disuse but that is still used daily in this location. People complained that some men from these families, and managers of these goods, wasted water only as a sample of what they could do, as a demonstration of power. Even if they did not use it, they were not willing to share.

In this sequence, one of the strategies that they want to implement is the introduction of a management and, consequently, a payment system for water usage in an attempt to show that the water is a public good, not private, even in the case of spring water, which is the Charco’s case. Hence the most recent negotiations with the National Water and Sanitation Agency (ANAS), which places on the horizon of these women the possibility of agricultural production.

Loide, a 58 year-old woman, has always shown herself strong and willing to work on her land. She tells us that she started to work from a very young age – "a child"; her parents did not enroll her in school, so she grew up in the daily toil. The rainy season was about to start, and there she was, coming and going, every day until the harvest. Even today, when the harvest period starts, she works on someone else's land and pays rent; and about half of the products harvested go straight to her tenant. She has the support of her husband and two of her seven children in this day-to-day life.

As Loide says, it would be enough to give them work for them to work. But at the same time, she asks: "Is it because we are women?". And she replies: “But we have someone to work with, we have children, we have a husband too. We are looking for work. To have water, it is the water ,that is missing!”

Loide’s testimonial is interesting to understand how the logic of concentration and exploitation of goods–land and water – , and the consequent power exercised, is masculine, making women live in inequality. An inequality that, at first glance, affects not only gender because it calls into question the women’s ability to make decisions regarding their subsistence means, but because it seems to run through social classes and hit men. Even when it is not them that are directly working, or being represented by their sons and husbands, who are also men, they recognize that there is no room for them.

Along the way, there are more women and families from the Charco in a similar situation, which instigates countless reflections. They express an immense desire to work and overcome difficulties, to have means, and to produce on their piece of land, losing the dependence on both their homes and their large landowners and tenants:

If they [the landowners] make *grogue* ^8^ it is for us to buy, if they make honey, we must buy, even the children cannot have a tip of cane! My children were raised and didn't go near the *fornadja* ^9^; I wouldn’t let them go because when they did, they would censure them saying that they would punch us. So, I raised them so that now they are grown, and are already someone. (Field notes, Loide, Charco, 04/2015).

However, this community resident raises, in addition to water, her concern and anxiety to install an alternative system to access water through a hole that needs an extra mechanism to work. In the medium and long term, the effects would be positive, but it was too expensive to install the drop by drop irrigation system, even though her share was small. Other women and her expected the help from the former Ministry of Rural Development, now the Ministry of Agriculture and Environment. Loide explains that,

They say they are going to help us, that they are going to install a pump in a hole to draw water, and that this will be used for irrigation, allowing us to work. Because if we find water, we will work even a little bit, piece by piece, and when we realize, we will have already finished. (Field notes, Loide, Charco, 04/2015).

Thus, Loide talks about a modernization scenario concerning agriculture which was put into motion some time ago, as mentioned by Victor (Reis, 2015, p. 152):

“The Cape Verde government has been allocating important resources to agriculture since the early years of Independence. To this end, it has counted on international help in both financial and technical fields.

The National Agricultural Investment Program, initiated in 2010, based on a six-year execution plan, foresees investments, some already underway, worth US$ 250 million to modernize agriculture. The expected funding is supported either by the Government (15.5%) or by external sources, namely the Portuguese Line of Credit (11.7%) and the BADEA^10^ (10.5%).

About 61% of this amount is intended to improve water management, being 52% used for the construction of dams, drilling holes, wells, dikes, pumping systems, desalination units, reservoirs, and 9% for the promotion of irrigation, in particular, micro-irrigation. Another important part (23%) is aimed at changing agricultural, forestry, and pastoral practices.”

The water above-mentioned by Donan, from the Brianda Norte Association, came to fall in reservoirs spread across the Charco. This water flowed through the rocks and poured into a gallery, built for this purpose in the colonial period. It was a disputed water and, as Loide reinforced to us, a water that the former landowners believed to own. Thus, Loide and the other women would have to wait for the pump installation to be able to access it through the hole that had been opened recently, even though against the will of the large landowners.

They have always had their water here [since] ancient times. It is older than my father, than my grandparents. So, now for us to get water from there, it’s tiring. Their water doesn't work anymore They think that because they had vegetable gardens at first, they are the owners. And us, who had just recently received these installments of land, had received it against the landowners will So, there is nothing left to us at all.
They are the greatest people of ancient times, and their water cannot be irrigated to this side and shared with us. They can't share it with us, so they don't do it. (Field notes, Loide, Charco, 04/2015).

In effect, the message sent by the Charco’s great landowners to these women was that they would always live without water and would get used to it. Unlike them, who always had their pots full and whose pots would always remain that way.

From these women’s perspective, not even the pieces of land distributed by the Ministry were subject to any satisfaction on behalf of the landowners–once landless, forever, then, they should be. Even if these women lands had been in the riverside, a space in which traditionally these landowners would never be interested in, either because it was a rainy path or because it was considered a State property. In their perceptions, they would have greater rights over both land and water, insofar as everyone, from those who live to those who had died, based on their circumstances and histories, was testimonies of their heritage antiquity.

At this moment, while waiting to see some outcome for their situation, these women looked at the sky willing to read the signs that could indicate a good agricultural year. The volcanic eruption on Fogo Island, in November 2014, was a good omen for the community, since, according to oral memory, in the years of eruptions there was always harvest in abundance. Although *azágua* ^11^ did not provide them with much food, at least there would be a lot of straw for their animals. Basically, what they had to do was dedicate themselves to plant every year, without losing hope, and counting that, if that year *azágua* was weak, God would allow that in the next it would be better.

### Final Considerations

The objective that led us to Ribeira da Barca and the Charco was to understand the women who worked in the sand harvesting, known for being blamed for the environmental degradation. According to Roberto Cardoso Oliveira, (2000), regarding the State’s actions, the ethical commitment to guarantee a symmetrical dialogue is a responsibility of the dominant pole, in this case, the State itself. Following the ethnicity proposal concerning an interethnic dialogue, as presented by Oliveira, (2000) when analyzing the State's actions directed at indigenous peoples in Brazil, we were guided by the commitment and the need to look at the sand extraction phenomenon from the women’s point of view.

While in the field immersion, we learned that the sand extraction is not a simple natural resource available or an occasional job, as one of the young men on the way to the Charco warned us; it is part of the Cape Verdean rural environment and is related to different dynamics, temporalities, memories, and interactions, considering human and non-human. We deliberate here based on Tim Ingold (2012, p. 32) idea of a “mutual permeability and connectivity” that shows the environment as an “open world” made from practices that are in constant movement or fluidity. For him, "things are alive, because they leak" (Ingold, 2012, p. 32), i.e., the boundaries between nature and culture are blurred. This is how we understand Mr. Sogni's knowledge regarding the sea and the rhythms of life in Ribeira da Barca and the Charco, but also regarding the hope of a good harvest, based on the message delivered by the volcano in Fogo, or concerning a sense of human limitations, from the recognition and sense of justice credited to the divine. Would this intimate dialogue with nature (and with non-humans) be a space cultivated and made possible by the colonial experience?

Concerning the sand harvesting, we observe that women are blamed, without, however, problematizing the inequalities structure in terms of gender and class. The ownership and control of resources–land and water–being a male domain demonstrates that this rural community inspires and exhales inequalities.

The “white scarf on the head” manifest, mentioned at the beginning of this text, establishes some historical links in the field of gender relations in Santiago. Wives of men who emigrated and remain in the country, maintaining a long-distance relationship, are recognized as “white scarf widows”, in opposition to the black scarf widows that precede a mourning state. This is shown in a study carried out by Veiga (2016) in the community of Pilão Cão, in São Miguel municipality. The author concludes that these women are fundamental pieces to the migration project of absent husbands, either due to the burden of social and economic maintenance of families, or because they are a link with other family members. Her study presents us with the social burden carried by these women who remain and are constrained to live as “white scarf widows”. Could the “white scarf on the head” manifest, organized by these women who are in the sand harvesting, be taken as a criticism to the gender system presented as a form of social organization and also in the body of the State?

## Data Availability

The raw data supporting the conclusions of this article will be made available by the authors, without undue reservation.

## Footnotes

1
*Batuko* is a traditional Cape Verdean manifestation, especially from Santiago Island. According to (Semedo, 2009, p. 14), “*Batuko* is mainly a feminine space, in which women, *batukadeiras*, through lyrics talk about men and women (about themselves and others), talk about the local and Cape Verdean space”.

2 The same criticism appeared in protected areas of Cape Verde, on Santiago and on São Nicolau Islands, among women who joined empowerment programs, and sought for credit to pig breeding; however, these women found themselves in debt since they went through this experience of not being able to feed the pigs, which made them displace family and the children’s education resources to keep up the enterprise and pay off the debts (Moassab and Vieira, 2016).

3 “The tide follows the moon", Mr. Sogni told us on March 7, 2010.

4 Very common large scarf or cloth used among women to overlay clothing, but which can have multiple uses, such as tying on the hips to increase balance, to give strength, to protect from the sun, etc.

5 Shovel used to extract sand.

6 These fisheries agreements with the European Union started in 2007 and continue being renovated. They allow vessels from Spain, Portugal, and France to fish in Cape Verdean waters. Based on an online news, it was possible to follow several demonstrations against the agreement renewal, among them: “Slavery continues, we are tired of working and we don’t gain anything from these Europeans here stealing our wealth”; “Europeans and China are destroyers of the seabed and trees. Then, in their land, Cape Verdeans are treated like thugs and slaves”; "Later on, we will start to buy tuna cans from the European Union". See in: http://www.asemana.publ.cv/spip.php?article93865.

7 Interview conducted in February 2010.

8
*Aguardente* made from sugarcane.

9 Space for the *grogue* production, *aguardente* made from sugarcane.

10 Arab Bank for Economic Development in Africa.

11 Water time, i.e., the rainy season in which irrigated agriculture is dedicated and runs from July to September.
